# Attitudes towards gambling, gambling participation, and gambling-related harm: cross-sectional Finnish population studies in 2011 and 2015

**DOI:** 10.1186/s12889-017-4056-7

**Published:** 2017-01-26

**Authors:** Anne H. Salonen, Hannu Alho, Sari Castrén

**Affiliations:** 1 Department of Tobacco, Gambling and Addiction, National Institute for Health and Welfare, P.O. Box 30, FI-00271 Helsinki, Finland; 2 Institute of Clinical Medicine, University of Helsinki, Helsinki, Finland

**Keywords:** Attitudes, Cross-sectional, Gambling, Gambling-related harm, Population study

## Abstract

**Background:**

Information about public gambling attitudes and gambling participation is crucial for the effective prevention of gambling-related harm. This study investigates female and male attitudes towards gambling, gambling participation, and gambling-related harm in the Finnish population aged 15–74.

**Methods:**

Cross-sectional random sample data were collected in 2011 (*n* = 4484) and 2015 (*n* = 4515). The data were weighted based on gender, age and region of residence. Attitudes were measured using the Attitudes Towards Gambling Scale (ATGS-8). Gambling-related harms were studied using the Problem Gambling Severity Index and the South Oaks Gambling Screen.

**Results:**

Attitudes towards gambling became more positive from 2011 to 2015. Female attitudes were generally negative, but nonetheless moved in a positive direction except in age groups under 25. Occasional gambling increased among women aged 18–24. Women aged 18–24 and 45–54 experienced more harms in 2015 than in 2011. Both land and online gambling increased among women aged 65–74. Male attitudes towards gambling were generally positive, and became more positive from 2011 to 2015 in all age groups except 15–17. Weekly gambling decreased among males aged 15–17. Gambling overall increased among males aged 18–24. Gambling several times a week decreased among men aged 35–44 and 45–54, and gambling 1–3 times a month increased in the latter age group. Online gambling increased only among men aged 55–64.

**Conclusions:**

Attitudes towards gambling became more positive in all except the youngest age groups. Under-age male gambling continued to decrease. We need to make decision-makers better aware of the continuing growth of online gambling among older people and women’s increasing experiences of gambling-related harm. This is vital to ensure more effective prevention.

## Background

Gambling opportunities have increased significantly in the past two decades, and at the same time gambling-related harm has grown into a public health concern and social issue worldwide. In many countries including Finland [1], Australia [2] and the UK [3], public attitudes towards gambling tend to be negative, more so among women than men [1, 3–5]. Male gender as well as age between 18 and 54 have been found to correlate with more positive attitudes towards gambling [1, 3], but some evidence indicates that age has no effect [6]. It has been reported that men and younger individuals typically gamble more and have a higher risk of developing gambling problems [7–11]. In Finland where this study was conducted, the national gambling monopoly has recorded growing profits since 2009. The monopoly’s revenue figures are among the highest in the EU [12]. Most of these profits are channelled through the state or NGOs to promote the public good.

According to the theory of planned behaviour (TPB) [13, 14], behaviours such as gambling participation are mediated by an individual’s attitudes, subjective norms and perceived behavioural control [6]. Positive attitudes towards gambling correlate with a high gambling frequency [1, 15]. On the other hand, experiences of gambling problems create more negative attitudes [2, 3, 16–19]. Epidemiological studies have shown that increased gambling participation, and higher gambling frequency in particular, leads to an increase in gambling problems [20, 21]. It seems that online gambling contributes more strongly to gambling problems than land-based gambling [22–24].

Gambling can also bring about different types of harms [25, 26]. The risk of individual harm is highest among problem gamblers, yet most gambling harms are also found among low-risk gamblers [25]. To better understand these phenomena, it is important to explore the occurrence of gambling harms across all levels of participation [25–28]. However, there are hardly any tools available to measure these harms at the population level. Previous population-based studies of gambling-related harm are limited to a restricted number of items derived from problem gambling instruments such as the Problem Gambling Severity Index (PGSI) (e.g. [25, 28]).

Public attitudes can provide importance guidance for governments as they seek to develop responsible gambling policies [2]. Analyses of gambling attitudes and gambling participation are therefore crucial tools that can help minimise gambling-related harm through gender- and age-specific prevention and treatment programmes.

This study compares attitudes towards gambling, gambling participation and gambling-related harm in Finland in 2011–2015, separately for men and women and different age groups. In addition, we use PGSI and the South Oaks Gambling Screen (SOGS) to produce a more comprehensive profile of gambling-related harm.

## Methods

The data for this study came from two cross-sectional Finnish gambling surveys in 2011 [29] and 2015 [30, 31], which drew random samples of 16,000 and 7400 people, respectively, from the population information register. The inclusion criteria were: 1) 15–74 years, 2) mother tongue Finnish or Swedish and 3) resident in mainland Finland. The exclusion criteria were: 1) living in an institution, 2) residing outside Finland (including Åland Islands) and 3) mother tongue other than Finnish, Swedish or Sami. In 2011, the study was described to the potential participants as a ‘gambling and health survey’, and in 2015 as a ‘gambling opinions and gambling survey’.

The data were obtained using computer-assisted telephone interviews. In 2011, a landline or mobile phone number was available for 11,129 respondents. An additional 120 phone numbers were determined by sending mail invitations to 4870 participants without a phone number. It turned out that 757 phone numbers were invalid. A further 1724 respondents could not be reached after a maximum of 10 attempts, while 4279 people refused to participate. Five respondents discontinued the interview after it had begun [1]. In 2011, 4484 interviews were completed, giving a response rate of 40% of eligible subjects [29].

In 2015 [30], 103 persons in the gross sample were not eligible (dead, permanent disability or illness, living abroad, permanently institutionalized). The number of eligible subjects was 7297, and 4515 interviews were completed, giving a response rate of 62%. The reasons for the attrition of 1594 persons (22%) were that 1125 had no phone number, 469 could not be reached, 275 avoided contact with the interviewer, 896 refused to participate and 17 other reasons. In both datasets, the most under-represented age group were respondents aged 15–34, while the most over-represented age group were respondents aged 65–74 [29, 30]. In 2015, the male response rate (62.4%) was slightly higher than the female rate (61.4%) [30]. Each interview lasted around 18 min. The data were weighted based on age, gender and region of residence (Northern, Eastern, Southern and Western Finland) in accordance with Statistics Finland’s national population-based registers in 2011 and 2015 [29, 30].

### Participants

Two demographic correlates were drawn from the population register: the respondent’s sex (male, female) and age, which was recoded into seven groups (15–17, 18–24, 25–34, 35–44, 45–54, 55–64 and 65–74). In 2011 and 2015, there were 2367 (Mean 44.5; SD = 16.6 years) and 2210 (Mean 45.6; SD = 17.0 years) female respondents aged 15–74, respectively. The corresponding figures for men were 2117 (Mean 43.8; SD = 16.6 years) and 2305 (Mean 44.8; SD = 16.8 years).

### Attitudes towards gambling

Attitudes were measured with the 8-item version of the Attitudes Towards Gambling Scale (ATGS-8) [4]. ATGS-8 items were scored using a Likert scale: 1 = “strongly agree”, 2 = “agree”, 3 = “neither agree nor disagree”, 4 = “disagree” and 5 = “strongly disagree”. Four items were reversely scored. The sum of items forms the total ATGS-8 score (range 8–40) where a score of 24 represents an overall neutral attitude towards gambling, while scores above 24 indicate a favourable (positive) and those below 24 an unfavourable (negative) attitude (Table 1). In 2011, the Finnish version of ATGS-8 reached an alpha value of 0.71 and factor analysis supported the use of two factors [1], which was consistent with findings based on the original 14-item instrument [16]. In 2015, the ATGS-8 reached an alpha value of 0.73.

**Table 1:** Female and male attitudes towards gambling by age in 2011 and 2015

	Females			Males		
	2011 Mean (95% CI)	2015 Mean (95% CI)	F_(df)_ [p; *η* ^2^]	2011 Mean (95% CI)	2015 Mean (95% CI)	F_(df)_ [p; *η* ^2^]
All	21.44 (21.18–21.69)	22.93 (22.71–23.16)	73.99_(1, 3978)_ [.001; .018]	23.97 (23.72–24.23)	25.22 (24.99–25.45)	50.81_(1, 4029)_ [.001; .012]
Age						
15–17 years	20.87 (19.66–22.08)	21.32 (20.32–22.33)	0.33_(1, 164)_ [.564; .000]	23.53 (22.07–24.98)	23.32 (22.14–24.50)	0.05_(1, 154)_ [.824; .000]
18–24 years	21.74 (21.06–22.43)	22.55 (21.95–23.16)	3.07_(1, 458)_ [.081; .007]	24.30 (23.64–24.95)	25.86 (25.23–26.49)	11.47_(1, 514)_ [.001; .021]
25–34 years	22.67 (22.07–23.27)	24.28 (23.74–24.82)	15.54 _(1, 664)_ [≤.001; .023]	25.38 (24.79–25.97)	26.40 (25.86–26.93)	6.25_(1, 674)_ [.013; .009]
35–44 years	21.38 (20.75–22.01)	23.53 (22.92–24.15)	22.57 _(1, 631)_ [≤.001; .035]	24.52 (23.91–25.13)	25.82 (25.27–26.36)	9.68_(1, 652)_ [.002; .014]
45–54 years	21.85 (21.24–22.47)	23.30 (22.73–23.87)	11.42 _(1, 678)_ [.001; .017]	24.38 (23.82–24.94)	25.21 (24.65–25.77)	4.13_(1, 718)_ [.042; .006]
55–64 years	20.70 (20.13–21.28)	22.41 (21.90–22.93)	19.02 _(1, 789)_ [≤.001; .024]	22.56 (21.98–23.15)	24.77 (24.22–25.32)	28.83 _(1, 773)_ [≤.001; .035]
65–74 years	20.05 (19.32–20.78)	22.02 (21.48–22.55)	17.94 _(1, 578)_ [≤.001; .030]	22.44 (21.67–23.21)	23.85 (23.26–24.44)	8.06_(1, 530)_ [.005; .015]

A one-way between-subject ANOVA (F-test) design for weighted data based on gender, age and region of residence; data in 2011 (*n* = 2367 females and *n* = 2117 males, non-weighted) and 2015 (*n* = 2210 females and *n* = 2305 males, non-weighted); 95% CI, 95% confidence intervals. Estimate of effect size (*η* ^2^ = eta-squared); The sum of 8 Attitudes Towards Gambling (ATGS-8) items (a Likert scale: 1 = “strongly agree”, 2 = “agree”, 3 = “neither agree or disagree”, 4 = “disagree” and 5 = “strongly disagree”, 4 reversed items) forms a total ATGS-8 score (range 8–40) < where a score of 24 represents the overall neutral attitude towards gambling, while scores above 24 indicate favourable and those below 24 unfavourable attitudes

### Gambling participation

Past-year gambling frequency (no gambling, less than monthly, 1–3 times/month, once a week, several times a week) and online gambling (yes/no) were examined using categorical variables.

### Gambling-related harms

In 2011 and 2015 gambling-related harm was measured using PGSI [32] and SOGS [33, 34], the strengths and limitations of which have been extensively reviewed in the literature (e.g. [7, 32–37]). Responses to the 9 PGSI items were on a four-point scale (0 = never, 1 = sometimes, 2 = most of the time, 3 = almost always). For the purposes of this study, the PGSI items were recoded to indicate the presence of harm (yes = scores 1–3) or the absence of harm (no = score 0). Responses to the 20 SOGS items were on a two-point scale (0 = no, 1 = yes). Next, duplicate items were combined (answer ‘yes’ to either the PGSI or the corresponding SOGS item), which yielded a total of 16 different harms that were included in the analysis (Table 2; items #2, #3, #5, #6). Furthermore, items related to borrowing money were combined into one (#12). The sum of the score of harms (range 0–16) was recoded to indicate experiencing no harm (score = 0), one harm (score = 1) or two or more harms (score ≥ 2). Non-gamblers were separated into their own group. This type of classification has been used in previous studies [25, 28]. A 12-month time frame was adopted to reflect current harms.

**Table 2:** Percentage of respondents reporting gambling-related harms measured using PGSI and SOGS in 2011 and 2015

		Females				Males			
Source		2011	2015	*p*	*ϕ*	2011	2015	*p*	*ϕ*
SOGS #4	1. Gamble more than you intended to	6.7	10.1	≤.001	.060	15.2	17.0	.123	.024
PGSI #1 / SOGS #1	2. Go back another day to win back money (chasing)	5.1	5.1	.944	.001	12.0	10.1	.035	−.033
PGSI #9 / SOGS #6	3. Felt guilty	2.8	6.4	≤.001	.085	7.0	7.4	.637	.007
PGSI #4	4. Need to gamble with larger amounts of money to maintain excitement	1.4	1.5	.898	.003	4.8	4.8	.943	.001
PGSI #7 / SOGS #5	5. People criticized your gambling	1.4	1.2	.496	−.011	3.6	3.3	.616	−.008
PGSI #2 / SOGS #3	6. Feel you have a problem	0.8	1.6	.024	.035	3.4	4.4	.117	.025
PGSI #3	7. Betting more than can afford to lose	1.3	1.7	.315	.016	4.2	4.2	1.000	.000
SOGS #7	8. Felt like you would like to stop gambling but didn’t think you could	1.2	2.4	.004	.044	2.1	2.4	.535	.011
SOGS #2	9. Claimed to be winning money gambling but weren’t really	0.9	0.4	.061	−.030	3.8	1.5	≤.001	.074
SOGS #9	10. Money arguments centred on gambling	0.8	1.2	.173	.022	1.3	1.5	.975	.005
PGSI #6	11. Gambling causing health problems	0.5	0.4	.667	−.007	1.6	2.0	.427	.014
PGSI #5 / SOGS #12-20	12. Borrowing money or selling something to finance gambling	0.4	0.5	.819	.007	1.0	0.7	.185	−.020
PGSI #8	13. Gambling causing financial problems	0.2	0.4	.588	.010	1.2	1.1	.772	.006
SOGS #11	14. Lost time from work or school	0.4	0.4	1.000	.000	0.8	0.9	1.000	.002
SOGS #8	15. Hidden betting slips	0.2	0.7	.046	.033	0.7	0.8	.604	.009
SOGS #10	16. Borrowed money and not paid them back	0.2	0.5	.221	.020	0.3	0.6	.249	.021

Significance (*p*) between time is determined by Fisher’s exact tests for weighted data based on gender, age and region of residence; Estimate of effect size (*ϕ* = phi coefficient); data in 2011 (*n* = 2367 females and *n* = 2117 males, non-weighted) and 2015 (*n* = 2210 females and *n* = 2305 males, non-weighted); *SOGS* the South Oaks Gambling Screen, *PGSI* the Problem Gambling Severity Index

### Data analysis

Two datasets were combined and a new variable reflecting the year was created. The data were analysed with SPSS version 22.0 (SPSS, Chicago, IL, USA). Before proceeding with the data analysis, variables were screened for possible outliers and statistical assumption violations with SPSS Frequencies, Explore and Plot procedures. We did not detect univariate outliers that were considered to require deletion. The estimates of skewness, kurtosis and normal probability plots did not indicate significant deviations from normality either [38]. Mean differences were analysed by between-subjects ANOVA designs. Pearson’s Chi-squared test and Fischer’s exact test were used for categorical variables. All comparisons were performed for different age groups between times within genders. 95% confidence intervals (CI) were also estimated. In addition, eta-squared (*η* ^2^) was used for ANOVA’s, and phi coefficient (*ϕ*) and Cramer’s V (*ϕ*_*C*_) for categorical variables to measure the strength of the examined associations. Thresholds for *η*2 were as follows: small (0.01), medium (0.06) and large (0.13) [39].

## Results

### Attitudes towards gambling

**Fig. 1:**
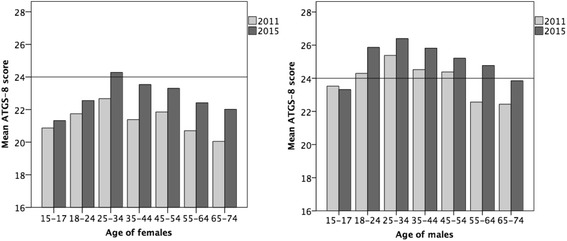
Attitudes towards gambling (ATGS-8) by gender and age in 2011 and 2015

Overall, attitudes towards gambling became more positive among both women and men aged 18–74 (*F* = 73.99, *p* = .001, *η* ^2^ = .018; *F* = 50.81, *p* = .001, *η* ^2^ = .012, respectively) from 2011 to 2015 (Table 1). In 2011, mean female ATGS-8 scores remained unfavourable (<24) in all age groups (Fig. 1). However, the mean scores of women aged 25–34 showed a change towards favourable attitudes (>24) in 2015. Female attitudes towards gambling were more positive in 2015 than in 2011 in all age groups (*p* ≤ .001, *η* ^2^ = .017–.035) except 15–17 and 18–24 (Table 1). Among males, the mean scores of those aged 18–54 showed a favourable attitude in 2011, but in 2015 men aged 55–64 also had a positive attitude towards gambling (Fig. 1). Furthermore, male attitudes towards gambling became more positive (*p* ≤ .05, *η* ^2^ = .006–.035) in 2015 in all age groups except 15–17.

### Past-year gambling participation

Past-year gambling frequency did not change statistically significantly from 2011 to 2015 among women aged 18–74. Among men, it did (*X* ^*2*^ = 17.272, df = 4, *p* = .002, *ϕ* = .064). There was an increase in the proportion of males who gambled 1–3 times a month (*p* ≤ .001). Women who gambled less often than monthly increased (*p* ≤ .005) in the age group 18–24, while non-gambling decreased (*p* = .009) among those aged 65–74 (Table 3). Among males aged 15–17, both gambling once a week (*p* = .021) and several times a week decreased (*p* ≤ .001), while non-gambling decreased among those aged 18–24 (Table 4). In the age groups 35–44 and 45–54, too, gambling several times a week decreased (*p* ≤ .05). Gambling 1–3 times a month increased in the latter age group (*p* ≤ .001).

**Table 3:** Past-year gambling participation and harms among females by age in 2011 and 2015 (% ± CI)

	15–17		18–24		25–34		35–44		45–54		55–64		65–74	
	2011 (*n* = 64)	2015 (*n* = 83)	2011 (*n* = 228)	2015 (*n* = 172)	2011 (*n* = 278)	2015 (*n* = 328)	2011 (*n* = 335)	2015 (*n* = 305)	2011 (*n* = 447)	2015 (*n* = 359)	2011 (*n* = 564)	2015 (*n* = 491)	2011 (*n* = 451)	2015 (*n* = 472)
Gambling frequency														
No gambling	66.7 ± 11.6	71.9 ± 9.7	32.2 ± 6.1	27.0 ± 6.6	21.0 ± 4.8	21.6 ± 4.5	24.4 ± 4.6	24.1 ± 4.8	19.7 ± 3.7	22.0 ± 4.3	23.8 ± 3.5	18.3 ± 3.4	36.2 ± 4.4	26.4 ± 4.0
Less than monthly	25.9 ± 10.7	20.8 ± 8.7	33.5 ± 6.1	42.2 ± 7.4	39.3 ± 5.7	39.3 ± 5.3	35.1 ± 5.1	36.7 ± 5.4	27.8 ± 4.2	30.6 ± 4.8	20.3 ± 3.3	25.2 ± 3.8	16.4 ± 3.4	18.5 ± 3.5
1–3 times/month	4.9 ± 5.3	5.2 ± 4.8	23.4 ± 5.5	19.3 ± 5.9	24.9 ± 5.1	24.4 ± 4.7	20.9 ± 4.4	19.6 ± 4.5	19.5 ± 3.7	19.9 ± 4.1	19.6 ± 3.3	17.9 ± 3.4	9.3 ± 2.7	14.4 ± 3.2
Once a week	1.2 ± 2.7	1.0 ± 2.1	6.7 ± 3.3	8.6 ± 4.2	11.7 ± 3.8	11.5 ± 3.5	17.4 ± 4.1	15.7 ± 4.1	27.0 ± 4.1	22.6 ± 4.4	29.4 ± 3.8	32.0 ± 4.1	32.8 ± 4.3	31.3 ± 4.2
Several times a week	1.2 ± 2.7	1.0 ± 2.1	4.2 ± 2.6	2.9 ± 2.5	3.0 ± 2.0	3.1 ± 1.9	2.2 ± 1.6	3.9 ± 2.2	5.9 ± 2.2	4.8 ± 2.2	6.9 ± 2.1	6.6 ± 2.2	5.2 ± 2.1	9.4 ± 2.6
Significance	*X* ^*2*^ = .699, df = 4, *p* = .952, *ϕ* = .063		*X* ^*2*^ = 5.677, df = 4, *p* = .225, *ϕ* = .108		*X* ^*2*^ = .056, df = 4, *p* = 1.000, *ϕ* = .009		*X* ^*2*^ = 2.120, df = 4, *p* = .714, *ϕ* = .056		*X* ^*2*^ = 2.894, df = 4, *p* = .576, *ϕ* = .062		*X* ^*2*^ = 6.065, df = 4, *p* = .194, *ϕ* = .084		*X* ^*2*^ = 12.343, df = 4, *p* = .015, *ϕ* = .0.138	
No gambling		.513		.232		.853		.928		.470		.053		.009
Less than monthly		.476		.050		1.000		.688		.420		.104		.531
1-3 times/month		1.000		.268		.930		.701		.926		.541		.054
Once a week		1.000		.495		1.000		.603		.174		.416		.734
Several times a week		1.000		.469		1.000		.267		.520		.893		.052
Online gambling														
Yes	0	2.1	14.4	16.3	19.9	26.3	16.9	23.2	19.2	17.0	12.5	15.2	4.0	9.4
Significance	*p* = .549, *ϕ* = .084		*p* = .603, *ϕ* = .027		*p* = .056, *ϕ* = .076		*p* = .051, *ϕ* = .079		*p* = .499, *ϕ* = − .028		*p* = .315, *ϕ* = .038		*p* = .016, *ϕ* = .099	
Gambling harms^a^														
No gambling	66.7 ± 11.6	71.9 ± 9.7	32.2 ± 6.1	27.0 ± 6.6	21.0 ± 4.8	21.6 ± 4.5	24.4 ± 4.6	24.1 ± 4.8	19.7 ± 3.7	22.0 ± 4.3	23.8 ± 3.5	18.3 ± 3.4	36.2 ± 4.4	26.4 ± 4.0
No harms	18.5 ± 9.5	17.5 ± 8.2	46.2 ± 6.5	42.9 ± 7.4	64.0 ± 5.6	58.4 ± 5.3	65.6 ± 5.1	65.1 ± 5.4	69.5 ± 4.3	62.3 ± 5.0	61.5 ± 4.0	64.5 ± 4.2	54.1	59.7
One harm	9.9 ± 7.3	3.1 ± 3.7	13.0 ± 4.4	14.3 ± 5.2	10.8 ± 3.7	13.8 ± 3.7	8.2 ± 2.9	6.7 ± 2.8	7.6 ± 2.5	8.1 ± 2.8	9.5 ± 2.4	9.9 ± 2.6	7.1	8.1
≥2 harms	6.1 ± 5.9	7.2 ± 5.6	8.8 ± 3.7	15.1 ± 5.4	4.5 ± 2.4	5.9 ± 2.6	1.9 ± 1.5	4.2 ± 2.3	3.0 ± 1.6	7.3 ± 2.7	5.1 ± 1.8	6.8 ± 2.2	3.0	6.0
Significance	*X* ^*2*^ = 3.481, df = 3, *p* = .323, *ϕ* = .140		*X* ^*2*^ = 5.586, df = 3, *p* = .134, *ϕ* = .108		*X* ^*2*^ = 2.743, df = 3, *p* = .433, *ϕ* = .063		*X* ^*2*^ = 3.373, df = 3, *p* = .338, *ϕ* = .071		*X* ^*2*^ = 8.712, df = 3, *p* = .033, *ϕ* = .109		*X* ^*2*^ = 4.444, df = 3, *p* = .217, *ϕ* = .072		*X* ^*2*^ = 9.031, df = 3, *p* = .029, *ϕ* = .118	
No gambling		.513		.232		.853		.928		.470		.053		.009
No harms		1.000		.465		.138		.935		.044		.396		.171
One harm		.115		.693		.248		.466		.891		.909		.657
≥2 harms		1.000		.036		.494		.119		.012		.319		.092

Data in 2011 (*n* = 2367, non-weighted) and 2015 (*n* = 2210, non-weighted); Significance (p) between time is determined by Chi-squared and Fisher’s exact tests for weighted data based on gender, age and region of residence; Estimate of effect size (*ϕ* = phi coefficient/Cramer’s V); ^a^Harms measured using 16 items from the South Oaks Gambling Screen and the Problem Gambling Severity Index; CI, 95% confidence intervals

**Table 4:** Past-year gambling participation and harms among males by age in 2011 and 2015 (% ± CI)

	15–17		18–24		25–34		35–44		45–54		55–64		65–74	
	2011 (*n* = 75)	2015 (*n* = 82)	2011 (*n* = 233)	2015 (*n* = 227)	2011 (*n* = 252)	2015 (*n* = 306)	2011 (*n* = 253)	2015 (*n* = 368)	2011 (*n* = 369)	2015 (*n* = 415)	2011 (*n* = 512)	2015 (*n* = 480)	2011 (*n* = 423)	2015 (*n* = 427)
Gambling frequency														
No gambling	40.0 ± 11.1	52.3 ± 10.8	18.7 ± 5.0	11.5 ± 4.2	11.5 ± 3.9	11.0 ± 3.5	13.9 ± 4.3	10.2 ± 3.1	14.0 ± 3.5	11.0 ± 3.0	17.5 ± 3.3	15.9 ± 3.3	24.8 ± 4.1	21.4 ± 3.9
Less than monthly	12.5 ± 7.5	18.6 ± 8.4	16.0 ± 4.7	16.7 ± 4.9	17.8 ± 4.7	20.4 ± 4.5	14.8 ± 4.4	20.2 ± 4.1	15.1 ± 3.7	16.4 ± 3.6	11.6 ± 2.8	12.0 ± 2.9	12.4 ± 3.1	10.6 ± 2.9
1-3 times/month	11.3 ± 7.2	20.9 ± 8.8	33.5 ± 6.1	39.8 ± 6.4	30.5 ± 5.7	34.7 ± 5.3	26.5 ± 5.5	30.9 ± 4.7	17.8 ± 3.9	27.9 ± 4.3	17.5 ± 3.3	16.6 ± 3.3	12.0 ± 3.1	17.0 ± 3.6
Once a week	20.0 ± 9.1	7.0 ± 5.5	20.2 ± 5.2	19.3 ± 5.1	26.7 ± 5.5	24.2 ± 4.8	29.7 ± 5.6	29.6 ± 4.7	36.1 ± 4.9	33.5 ± 4.5	38.4 ± 4.2	37.1 ± 4.3	38.0 ± 4.63	34.9 ± 4.5
Several times a week	16.3 ± 8.4	1.2 ± 2.4	11.7 ± 4.1	12.6 ± 4.3	13.5 ± 4.2	9.7 ± 3.3	15.1 ± 4.4	9.1±	17.0 ± 3.8	11.3 ± 3.1	15.0 ± 3.1	18.4 ± 3.5	12.8 ± 3.2	16.1 ± 3.5
Significance	*X* ^*2*^ = 21. 221, df = 4, *p* ≤ .001, *ϕ* = .358		*X* ^*2*^ = 6.109, df = 4, *p* = .191, *ϕ* = .108		*X* ^*2*^ = 4.396, df = 4, *p* = .355, *ϕ* = .078		*X* ^*2*^ = 10.922, df = 4, *p* = .027, *ϕ* = .127		*X* ^*2*^ = 14.845, df = 4, *p* = .005, *ϕ* = .140		*X* ^*2*^ = 1.996, df = 4, *p* = .736, *ϕ* = .049		*X* ^*2*^ = 4.874, df = 4, *p* = .300, *ϕ* = .092	
No gambling		.122		.028		.906		.156		.579		.363		.066
Less than monthly		.292		.906		.395		.070		.691		.915		.505
1-3 times/month		.098		.148		.234		.204		≤.001		.783		.121
Once a week		.021		.827		.442		1.000		.447		.722		.427
Several times a week		p ≤ .001		.790		.129		.018		.028		.197		.339
Online gambling														
Yes	12.7	5.9	29.8	33.1	47.1	48.2	36.2	40.7	26.9	32.5	13.6	21.9	8.5	10.8
Significance	*p* = .239, *ϕ* = − .119		*p* = .448, *ϕ* = .035		*p* = .821, *ϕ* = .011		*p* = .235, *ϕ* = .046		*p* = .109, *ϕ* = .061		*p* = .003, *ϕ* = .107		*p* = .385, *ϕ* = .039	
Gambling harms^a^														
No gambling	40.0 ± 11.1	52.9 ± 10.8	18.7 ± 5.0	11.5 ± 4.2	11.5 ± 3.9	11.0 ± 3.5	13.8 ± 4.3	10.2 ± 3.1	14.0 ± 3.5	11.0 ± 3.0	17.4 ± 3.3	15.9 ± 3.3	24.8 ± 4.1	21.4 ± 3.9
No harms	21.0 ± 9.2	30.6 ± 10.0	33.5 ± 6.1	42.2 ± 6.4	54.2 ± 6.2	50.7 ± 5.6	63.0 ± 6.0	60.8 ± 5.0	60.9 ± 5.0	65.4 ± 4.6	61.2 ± 4.2	60.4 ± 2.4	60.3 ± 4.7	63.6 ± 4.6
One harm	12.5 ± 7.5	4.7 ± 4.6	24.9 ± 5.6	23.8 ± 5.5	15.5 ± 4.5	19.4 ± 4.4	11.9 ± 4.0	15.6 ± 3.7	13.0 ± 3.4	15.1 ± 3.4	14.0 ± 3.0	14.7 ± 3.2	10.2 ± 2.9	7.3 ± 2.5
≥2 harms	26.3 ± 10.0	10.6 ± 6.7	23.0 ± 5.4	22.2 ± 5.4	18.9 ± 4.8	18.8 ± 4.4	11.3 ± 3.9	13.3 ± 3.5	11.9 ± 3.3	8.5 ± 2.7	7.6 ± 2.3	8.8 ± 2.5	5.1 ± 2.1	7.3 ± 2.5
Significance	*X* ^*2*^ = 11.359, df = 3, *p* = .010, *ϕ* = .263		*X* ^*2*^ = 7.317, df = 3, *p* = .062, *ϕ* = .118		*X* ^*2*^ = 2.034, df = 3, *p* = .565, *ϕ* = .053		*X* ^*2*^ = 4.077, df = 3, *p* = .253, *ϕ* = .077		*X* ^*2*^ = 4.780, df = 3, *p* = .189, *ϕ* = .079		*X* ^*2*^ = 0.732, df = 3, *p* = .866, *ϕ* = .030		*X* ^*2*^ = 3.378, df = 3, *p* = .337, *ϕ* = .077	
No gambling		.122		.028		.906		.156		.579		.364		.072
No harms		.215		.039		.371		.580		.229		.832		.431
One harm		.095		.839		.171		.183		.406		.844		.227
≥2 harms		.014		.917		1.000		.484		.120		.615		.387

Data in 2011 (*n* = 2117, non-weighted) and 2015 (*n* = 2305, non-weighted); Significance (p) between time is determined by Chi-squared and Fisher’s exact tests for weighted data based on gender, age and region of residence; Estimate of effect size (*ϕ* = phi coefficient/Cramer’s V); ^a^Harms measured using 16 items from the South Oaks Gambling Screen and the Problem Gambling Severity Index; CI, 95% confidence intervals

Overall, past-year online gambling increased from 14.7 to 17.1% among females (*p* = .040) and from 27.2 to 30.1% among males (*p* = .036) between 2011 and 2015. However, a statistically significant increase in online gambling was only seen among women aged 65–74 (*p* = .016; Table 3) and men aged 55–64 (*p* = .003; Table 4).

### Past-year gambling-related harms

Past-year gambling-related harm increased among women aged 18–74 (*X* ^*2*^ = 17.391, df = 3, *p* ≤ .001). In 2011, 9.2% of female respondents experienced a single harm and 4.3% experienced two or more harms. These figures were higher in 2015: 9.7% experienced a single harm and 7.1% experienced two or more harms. In 2011, 14.6% of males experienced one harm and 13.3% two or more harms. The figures in 2015 were 15.3 and 12.6%. The differences were not statistically significant for males.

“Gambling more than one intended to”, “chasing losses” and “feeling guilty” were the three most common harms in both genders (Table 2). The proportion of females who endorsed the items “gambling more than one intended to”, “feeling guilty, “feeling that one has a gambling problem”, “feeling like you would like to stop gambling, but didn’t think you could”, “hiding betting slips”, all increased between 2011 and 2015 (*p* ≤ .005). However, the proportion of males who endorsed the items “chasing losses” and “claiming to be winning money gambling, but weren’t really”, decreased (*p* ≤ .005).

Age group analyses showed that the proportion of two or more harms increased among women aged 18–24 and 45–54 between 2011 and 2015 (*p* ≤ .005; Table 3). Among males, the proportion of two or more harms decreased among those aged 15–17 (*p* = .014), and the proportion of those aged 18–24 without any harms increased (*p* = .039) (Table 3). Further analysis of the 2015 data showed that “gambling more than one intended to” was the most common harm in all age groups and for both genders. However, “feeling guilty” was the second most common harm among all female age groups and among men aged 65–74, whereas “chasing losses” was the second most common harm for the other male age groups.

## Discussion

Attitudes towards gambling became significantly more positive in Finland from 2011 to 2015. Female attitudes, though, were still unfavourable: only women aged 25–34 took a positive view on gambling in 2015. Men aged 18–54 had a generally positive attitude in 2011, and by 2015 the age group 55–64 also took a positive view. Overall these results show a clear tendency towards more favourable gambling attitudes and towards a narrowing of gender differences – a major departure from earlier results in Finland, and from results in the UK and Australia [1, 3, 4, 16, 40].

The exceptionally positive attitudes that we found in comparison with the UK and Australia are probably explained by a complex interplay of several factors, such as gambling environment, gambling exposure, gambling types and gambling resources [41]. Perhaps most importantly, the gambling environment in Finland is one controlled by a government monopoly, which feeds back most of the profits from gambling operations to promote the arts and sciences, youth work, health care, research projects and other good causes.

Another possible explanation for the change in attitudes is the increased public exposure to gambling during the past decade [42, 43]. Today, there are some 20,000 EGMs in supermarkets, kiosks and petrol stations, and even pharmacies and hospital cafeterias across Finland. More work is needed to establish whether this kind of gambling exposure, and particularly the high density of EGMs that is a known risk factor for gambling-related harms [44, 45], have influenced public attitudes.

People in Finland are also exposed to gambling through marketing campaigns in which gambling operators are keen to emphasise that profits from gaming are used for good causes: “Gambling for the public good”, as one of the slogans says. This has been going on for decades and may well go a long way towards explaining the overall positive attitudes. The liberalisation and normalisation of gambling in general may also be conducive to more positive attitudes towards gambling. In general, in 2011 people in Finland tended to express their views more strongly than was in 2010 the case in the UK [3]. This may reflect a greater familiarity with the main ATGS-8 arguments, and could also be a result of the livelier public discussion and debate around gambling [2, 3].

Gambling frequency remained largely unchanged despite the change in attitudes, yet significant changes were observed within age groups. In a bid to protect young people from potential gambling-related harms, the Finnish government raised the gambling age limit from 15 to 18 years in 2010–2011. This immediately brought a reduction in the prevalence of gambling and problem gambling [18, 29, 46]. Our results show that under-age male weekly gambling continued to fall in 2011–2015. Furthermore, under-age female and male attitudes towards gambling remained unchanged, which may also be attributable to the law change.

The prevalence of problem gambling is typically highest among young males [7, 8, 30]. In 2007–2011, regular gambling in the age group 18–24 seemed to be decreasing [46]. Therefore, the changes we observed in this age group in 2011–2015 were somewhat surprising. That is, occasional gambling increased among women aged 18–24, and they also experienced more harm. This may help to explain why their attitudes did not become more positive: as was discussed earlier, experiences of gambling-related harm predict more negative attitudes [1, 16, 30]. Furthermore, gambling increased among men in this same age group. The results imply that as people reach legal gambling age, their experimentation with gambling seems to increase. Further efforts are needed to step up protection, prevention and harm reduction interventions among young people.

In older age groups, frequent gambling decreased among women aged 35–44 and 45–54, and occasional gambling increased in the latter age group. This latter trend was already seen in 2007–2011 [18, 46]. In 2015, women aged 45–54 experienced more harms than before. In addition, both land and online gambling increased among women aged 65–74. Previous studies indicate that older adults (50 or over) gamble less than younger adults [47]. On the other hand, older age brings several vulnerabilities: poor social adjustment and stressful life events, such as retirement and widowhood [48], physical, emotional and mental health issues [48, 49] and lack of support from social networks [50]. There is also evidence that neurobiological changes may increase gambling [51].

The growth of female gambling seems to be a fairly universal phenomenon [7, 52, 53]. There are indications that women are also more likely than men to be influenced by gambling advertisements and to play free games [54]. In Finland, the monopoly gambling operators have recently launched a range of female-friendly online games and so contributed to the gambling industry’s push to get larger numbers of older women to play online [55].

Women have been reported to regard the online internet platform as a safe place to gamble [56]. Online gambling is typically considered a domain of the younger male generation [57], but we found that it has also increased among men aged 55–64. This may reflect the growing interest in all age groups in limitless internet access through computers, mobiles, tablets and other wireless devices [58]. Statistics Finland data show that from 2011 to 2015, the proportion of internet users in Finland increased from 81 to 90% in the age group 55–64 and from 53 to 69% in the age group 65–74 [59].

The literature on older individuals’ online gambling as well as on gender differences in online gambling is scarce [60]. In general, we know that females tend to favour non-skill games such as slot machines and bingo [61]. These games are continuous forms of gambling in which the interval between betting and its outcome is very short and which enabled rapid and repeated gambling within a very short period of time [62, 63]. It has been argued that some older women begin to gamble more as their gendered caring role decreases [64]. It is clear that more research is needed into older individuals’ increased online gambling and into their motivations to gamble.

TPB provides one possible explanation for the trends we observed in Finnish women’s attitudes and participation in gambling [13, 14]. The theory suggests that the particular intention to gamble is influenced by positive attitudes (i.e. the perceived favourability of the outcome) and social norms (i.e. how a particular behaviour is approved by other people) [6]. TPB cannot, however, explain the trends seen among Finnish men: even though their attitudes became more positive, there was no change in their gambling participation or experienced gambling-related harms. As favourable attitudes towards gambling are associated with more frequent gambling, they may be considered a risk factor for gambling problems [3].

The question raised by the findings of our study is this: Have men in Finland now reached the point where the excitement and novelty value of gambling has begun to fade? Has male gambling reached saturation point at the same time as women are just beginning to join the bandwagon? Or is the growth of permissive attitudes an indication of an increased prevalence and intensity of gambling, as proposed by the total consumption model [65], a trend that will eventually lead to the normalisation of excessive gambling, especially among women? The potential feminisation of gambling should be recognised as a serious concern: women tend to start gambling later on in life, and gambling therefore develops into a problem more rapidly than in the case of men [66]. We need to continue to explore these potential gender-specific and socio-cultural connections.

In both males and females the three most common gambling-related harms in our study were “gambling more than one intended to”, “chasing losses” and “feeling guilty”. This is consistent with the findings of a previous Finnish study [25], which reported no gender differences in harm profiles. The harms that increased among the females in our study highlight the negative consequences to the individual gambler, such as “feeling that one has a gambling problem” and “feeling like you would like to stop gambling, but didn’t think you could”. In males, by contrast, both harm to the gambler himself and harm caused to others decreased.

It is noteworthy that SOGS and PGSI items measuring guilt and lending money produced quite widely differing results. Based on SOGS, 6.2% of the respondents reported feelings of guilt, while the corresponding PGSI figure was only 2.4% [30]. Furthermore, SOGS showed a slightly higher proportion lending money than PGSI (0.6% vs. 0.4%). In the questionnaire, the SOGS questions came before the PGSI items. The use of both PGSI and SOGS items allowed us to examine gambling-related harms more extensively than earlier studies in this field.

The conceptual framework of harmful gambling recently proposed by Browne et al. [67, 68] lists a broader range of gambling-related harms for gamblers, their significant others and the wider community. These dimensions of harm are: 1) financial harms, 2) relationship disruption, conflict or breakdown, 3) emotional or psychological distress, 4) detriment to health, 5) cultural harms, 6) reduced performance at work or study and 7) criminal activity [66, 67]. Furthermore, the framework identifies three temporal dimensions of experiencing harm: 1) general harm, 2) legacy and 3) crisis. General harm may occur at any point in time after engaging in gambling, while ‘legacy harm’ continues after the person’s gambling has stopped. Harms labelled as ‘crisis’, especially financial harms, typically trigger the motivation to seek help/treatment. Evidence from four countries indicates that player loss-risk curves for total gambling expenditure (losses) are likely to be linear or r-shaped [26]. More research is also needed on gambling expenditure and gamblers’ income [67], since it has been reported that gambling expenditure predicts gambling-related harm [26], and spending excessive amounts of money on gambling represents a risk factor for a variety of health outcomes [69].

### Study limitations

Our 2011 and 2015 datasets were collected by different organisations: the first by market research company Taloustutkimus, and the second by Statistics Finland. This may explain the differences in the response rates, which were below the national average in the first survey and over the average in the second. This adversely affects the comparability of our results [7]. Typically, high response rates in population studies tend to increase the proportion of infrequent gamblers [7, 70]. This may well have impacted our results, but it certainly cannot exhaustively explain the substantial shift observed towards more positive attitudes. Furthermore, although our sample sizes overall were quite large, the various subgroups were relatively small and therefore interpretations must be made with caution, especially in the age group 15–17. Many of the estimates we presented were not robust, since they were smaller than the lengths of the corresponding CIs. Overall, the effect sizes of the results were small to medium, implying that even though there were statistically significant group differences, the magnitude of these differences was not notable [38]. Our comparisons in this study were between two time points only, which is an obvious limitation, but on the other hand both studies were fairly similar in terms of methodology. Finally, we used PGSI and SOGS for purposes for which they were not originally intended. Nonetheless it is possible that the respondents may have experienced harms not measured by these instruments.

## Conclusions

With the exception of females aged 15–24 and males aged 15–18, attitudes towards gambling became more positive among Finnish women and men from 2011 to 2015. During this period, gambling participation increased most noticeably among females. Our findings for 2015 show for the first time an increase in gambling-related harm among females in Finland.

More research is needed on gambling and gamblers, especially women’s gambling motivations. Specific focus must be given to gambling-related harm and gambling-related factors, such as the gambling environment, gambling exposure and gambling types. This is crucial to developing more effective policy measures and to improving gambler protection, prevention and harm reduction efforts.

## Abbreviations

ATGS-8: the 8-item version of the Attitudes Towards Gambling Scale
EGM: Electronic gaming machine
PGSI: Problem Gambling Severity Index
SOGS: South Oaks Gambling Screen
TPB: Theory of planned behaviour
